# Acute Intermittent Porphyria’s Symptoms and Management: A Narrative Review

**DOI:** 10.7759/cureus.36058

**Published:** 2023-03-13

**Authors:** Esma Z Kizilaslan, Nitin M Ghadge, Andrea Martinez, Michelle Bass, Rahul Winayak, Midhun Mathew, Rutvi Amin, Muhammad Khan, Nadeem Kizilbash

**Affiliations:** 1 Department of Internal Medicine, Heinrich Heine University, Düsseldorf, DEU; 2 Department of Communicable Disease, New York State Department of Health, Albany, USA; 3 School of Medicine, Universidad Autónoma de Guadalajara, Zapopan, MEX; 4 School of Medicine, El Bosque University, Bogotá, COL; 5 Department of Surgery, Bristol Medical School, University of Bristol, Bristol, GBR; 6 Department of Internal Medicine, Pennsylvania Hospital, Philadelphia, USA; 7 Department of Health Sciences, Marie Curie Science Research Center, Greensboro, USA; 8 Department of Internal Medicine, Ayub Teaching Hospital, Abbottabad, PAK; 9 Department of Medical Laboratory Technology, Northern Border University, Arar, SAU

**Keywords:** abdominal pain, liver, aminolevulinic acid, porphobilinogen, hydroxymethylbilane synthase, heme, porphyrias, hemin, acute intermittent porphyria

## Abstract

Acute intermittent porphyria (AIP) is an autosomal dominant disorder of heme biosynthesis in the liver that is caused by the accumulation of toxic heme metabolites aminolevulinic acid (ALA) and porphobilinogen (PBG) due to a deficiency in the enzyme hydroxymethylbilane synthase (HMBS). The prevalence of AIP is found to commonly affect females of reproductive age (ages 15-50) and people of Northern European descent. The clinical manifestations of AIP include acute and chronic symptoms that can be outlined into three phases: the prodromal phase, the visceral symptom phase, and the neurological phase. Major clinical symptoms involve severe abdominal pain, peripheral neuropathy, autonomic neuropathies, and psychiatric manifestations. Symptoms are often heterogeneous and vague, which can lead to life-threatening signs if not treated and managed appropriately. Whether treating AIP in its acute or chronic form, the cornerstone of treatment consists of the suppression of the production of ALA and PBG. The mainstay of managing acute attacks continues to comprise discontinuing porphyrogenic agents, adequate caloric support, heme treatment, and the treatment of symptoms. In recurrent attacks and chronic management, prevention is key with the consideration of liver transplantation and/or renal transplantation. In recent years, there has been great interest in emerging treatments that focus on a molecular level such as enzyme replacement therapy, *ALAS1 *gene inhibition, and even liver gene therapy (GT), which has changed the way of traditionally managing this disease and will pave the way for innovative therapies to come.

## Introduction and background

**Figure 1:**
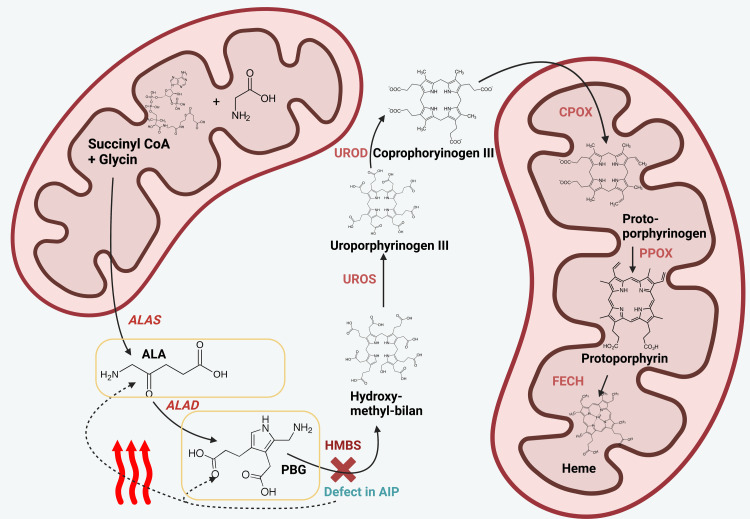
Pathomechanism of AIP. The figure shows the process of heme biosynthesis. Heme is a molecular precursor to hemoglobin and necessary for the subsequent binding of oxygen by mature erythrocytes. AIP is a rare autosomal dominantly inherited disorder of heme biosynthesis, in which different mutations can lead to the dysfunction of the enzyme HMBS (marked through a red cross). This can cause the accumulation of intermediary metabolites, such as ALA and PBG (framed in yellow rectangles). The buildup of these metabolites leads to diverse neurovisceral disorders. ALA and PBG accumulate and may be elevated in urine and plasma. This is a leading finding for the differential diagnostic workup of AIP. Created with BioRender.com ALA, aminolevulinic acid; ALAS, aminolevulinic acid synthase; ALAD, aminolevulinic acid dehydratase; PBG, porphobilinogen; HMBS, hydroxymethylbilane synthase; UROS, uroporphyrinogen III synthase; UROD, uroporphyrinogen decarboxylase; CPOX, coproporphyrinogen oxidase; PPOX, protoporphyrinogen oxidase; FECH, ferrochelatase; CoA, coenzyme A; AIP, acute intermittent porphyria

Acute intermittent porphyria (AIP) is a rare inherited metabolic autosomal dominant disorder that affects heme biosynthesis. It is characterized by a deficiency or defect of the hepatic enzyme hydroxymethylbilane synthase (HMBS), also known as porphobilinogen deaminase (PBGD), which is the third enzyme of the heme biosynthesis pathway (Figure 1) [1,2]. The first enzyme of the heme biosynthesis pathway is aminolevulinic acid (ALA) synthase-1 (ALAS1), which is also the rate-limiting enzyme that regulates heme storage. Any defect or deficiency of the heme biosynthesis pathway will cause an upregulation of ALA synthase-1 to overproduce and accumulate toxic heme precursors aminolevulinic acid (ALA) and porphobilinogen (PBG) in the liver [3,4].

The burden of AIP is significant, with a lifetime risk of one in 20,000 individuals [5]. The disease is more common in certain populations, such as individuals of Northern European descent, with a higher frequency in Sweden [5]. For this reason, it is also known as Swedish porphyria. Epidemiological studies on AIP have been conducted in various countries and show a wide range of prevalence rates. AIP, the most common acute porphyria, has an overall European prevalence of approximately one in 2,000, with a higher incidence of one in 1,000 in Sweden due to the founder effect. Recently, there are also reports of the founder effect correlating with a higher prevalence of AIP in certain ethnic groups in Argentina and Spain [5,6]. The difference in prevalence rates could be caused by differences in population genetics and how genetic testing is done.

AIP is a disease that affects both males and females and has a wide range of symptoms. Females are more seriously affected by AIP episodes in terms of frequency, attack duration, and the need for hospitalization [7]. Moreover, females have their first onset of disease at a younger age in comparison to males: A disease onset at an age of over 40 years was recorded by 18% of males in a Swedish study, whereas that rate was as low as 3% for females [7]. The majority of females (41%) had their first onset of disease at an age of 20-29 years [7]. For pediatric patients, there are only very few reports, and most of them are based on male pediatric patients [8].

In patients with AIP, the accumulation of ALA and PBG is caused by precipitating factors such as certain drugs, alcohol, infection, fasting, hormone fluctuations, or stress. The accumulation of ALA and PBG is responsible for the clinical manifestation of several symptoms. The most common clinical findings of AIP are severe abdominal pain, nausea, vomiting, weakness, constipation, and the classic darkened urine discoloration. AIP can lead to severe neurological symptoms (neuropathy and seizures) and psychiatric disturbances (anxiety, depression, and psychosis). Other chronic conditions, including hypertension, hyponatremia, kidney disorders, and even an increased risk of primary hepatocellular carcinoma, may arise in patients with severe AIP, which represents a growing lifelong burden for patients and their families [9,10].

The current diagnostic methods include measuring elevated porphyrin levels in the serum, urine, and stool [11,12]. The genetic analysis of HMBS mutations is recommended for all patients [11,12].

The management of AIP is multifaceted and involves the treatment of acute attacks, prevention of attacks, long-term monitoring, and treatment of related complications. For acute attacks, heme administration is recommended. Glucose may also be administered, if hemin is not readily available [11,12]. Heme prophylaxis can effectively control recurrence. The emergence of givosiran (hepatic ALAS1 targeting small interfering (si) RNA) represents great progress in treating AIP based on its etiology, and human porphobilinogen deaminase (hPBGD) messenger RNA (mRNA) is a promising treatment [11,12]. Liver transplantation is the last resort for patients with AIP [11,12].

Despite the progress that has been made in understanding AIP, there are still gaps in our understanding of the disease. For example, the exact mechanisms by which porphyrin precursors lead to the symptoms of AIP are not fully understood. Also, there is not a lot of long-term information about how well the current treatments work or how the disease progresses on its own. AIP is a neurovisceral disorder and one of the few porphyrias that can present with acute porphyria attacks, but despite this, the clinical presentation is nonspecific and widely variable, which has been shown to delay its diagnosis and management [1].

Our aim is to summarize the current state of evidence in the form of a narrative review, in order to give a needed insight into this rare condition for physicians, clinical practitioners, and students, as well as point out recommendations for further research on this field. We want to underline the significant research gap, portray scientific targets of interest for AIP management, and point out possible areas where further investigation is needed.

## Review

Symptoms

Phases of AIP: The Grouping Together of Symptoms

**Figure 2:**
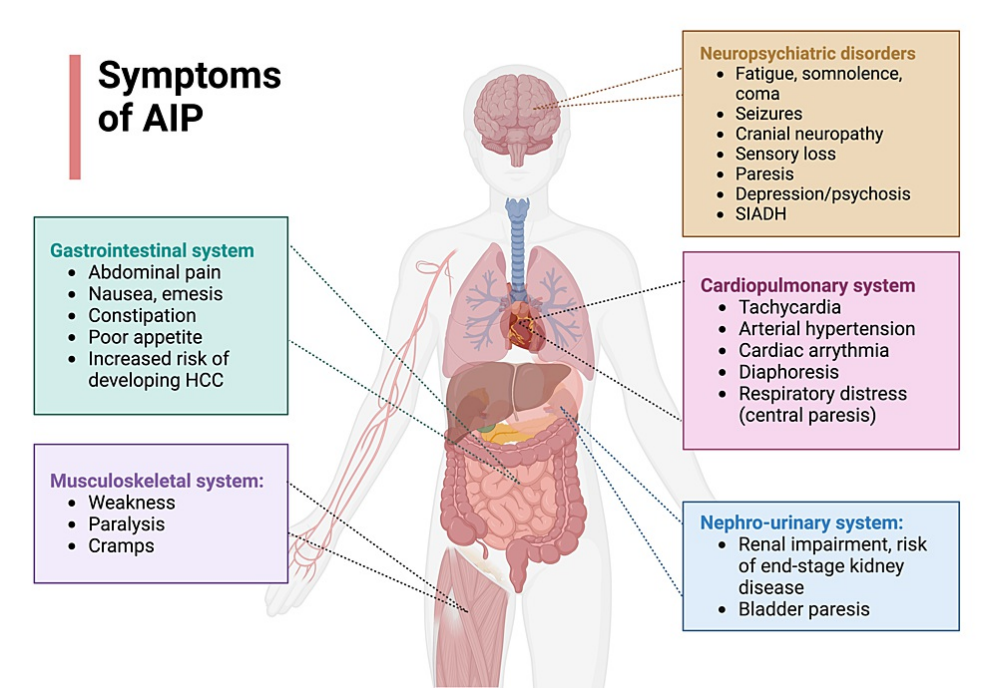
Overview of the diverse clinical manifestations of AIP. The most important gastrointestinal, neuropsychiatric, musculoskeletal, cardiopulmonary, and nephro-urinary symptoms are summarized. Adapted from “Glucose Transporters Distribution,” created by Gaia Lugano using BioRender.com (2023). Retrieved from https://app.biorender.com/biorender-templates AIP, acute intermittent porphyria; HCC, hepatocellular carcinoma; SIADH, syndrome of inappropriate antidiuretic hormone secretion

The symptoms of AIP usually occur in three phases: the prodromal phase, the visceral symptom phase, and the neurological phase [13]. Most of the patients initially experience rather unspecific prodromic symptoms such as nausea, emesis, fatigue, dizziness, weakness, headaches, and myalgias. Afterward, the visceral phase occurs, in which patients develop the main symptom of acute abdominal severe pain. Finally, the neurological phase may follow in which patients concomitantly develop neuropsychiatric symptoms, which are described in further detail below. In most cases of acute attacks of AIP, this pattern is observed, but deviations can also be seen. Patients may show a clinical heterogenous course. An overview of the diverse clinical manifestations of AIP is given in Figure 2.

Most of the patients with an acute porphyric attack present with severe abdominal pain without abdominal guarding [13]. Other common manifestations that have been described during acute attacks are peripheral neuropathy, autonomic neuropathies, and psychiatric manifestations [1]. The patient can have acute and chronic manifestations of AIP [14].

The neurological symptomsare due to the involvement of both the peripheral and central nervous systems [1]. Peripheral neuropathy can cause pain in the back and limbs, weakness/paresis, respiratory paresis, cranial neuropathy, and neuropathic sensory loss [15]. The central nervous system involvement causes seizures, coma, blurred vision, Babinski signs, nystagmus and cerebellar ataxia, posterior reversible encephalopathy syndrome (PRES), and syndrome of inappropriate antidiuretic hormone secretion (SIADH) [14-17]. A majority of AIP symptoms (e.g., abdominal vomiting and pain, constipation, excessive sweating, postural dizziness, and peripheral motor weakness) are due to a dysfunction of the autonomic nervous system [17,18]. Autonomic dysfunction seems to develop especially at the later stages of the disease; most of the affected patients have an established diagnosis of AIP for at least over 30 years [18]. Autonomic disorder associated with AIP has shown itself to be reversible, in contrary to other etiologies of autonomic dysfunction, such as diabetic neuropathy, familial dysautonomias (Riley-Day syndrome), or amyloidosis [18].

The psychiatric symptomsof an acute attack of AIP are very variable and include a wide spectrum of disorders. The symptoms include psychosis and catatonia. In addition, a wide range of disorders can be the presenting features, such as bipolar spectrum, depressive, anxiety, obsessive-compulsive, somatic symptom, disruptive, impulse control and conduct, neurocognitive, personality, and chronic fatigue disorders [15,19]. Sleep-wake disorders, including insomnia and restlessness, may occur too.

The most common gastrointestinal manifestation, abdominal pain, presents in over 80% of the patients [1]. Various nonspecific symptoms such as nausea, vomiting, and poor appetite are also associated with acute attacks [1]. It has long been known that the severe abdominal pain associated with AIP can often mimic acute intra-abdominal catastrophes such as those related to ischemic origin, and unnecessary surgical exploration is not uncommon [20,21]. On the other hand, due to acute abdominal manifestations in patients with porphyria, previous reports showed that mistakes of delayed lifesaving surgical procedures for such patients need urgent and essential interventions [22]. The patient also can develop progressive liver disease, cirrhosis, and hepatocellular carcinoma [1,14].

Cardiovascular manifestations are due to sympathetic nervous system activity and autonomic dysfunction and may cause tachycardia, arrhythmias, hypotension, and diaphoresis [1,18].

Renal impairmentis very common in patients with frequent attacks of AIP [14]. The resistant hypertension present in the patients with AIP also contributes to chronic kidney disease, and in most cases, the patient progresses to end-stage renal disease [1,14]. In one case-control study carried out in 50 AIP patients, hypertension was found to be more common in patients that clinically manifested AIP than those with latent AIP [23].

The endocrine system may be affected too. SIADH presents in about 25%-60% of patients with AIP, and patients develop hyponatremia. Patients can also develop hyperthyroidism, due to an increase in thyroid-binding globulin during an acute attack [1].

A thorough review of the literature has demonstrated that AIP is a heterogeneous disease in both its acute and chronic manifestations. In its acute presentation, although non-peritonitic abdominal pain is a common feature, this symptom is shared by several intra- and extra-intestinal conditions, such as gastroenteritis and pregnancy. In addition, when examined separately, the symptoms of AIP such as peripheral neuropathy, psychosis, and autonomic neuropathies may easily be attributed to other diseases. The overlapping symptoms of AIP with other diseases, in conjunction with the fact that patients with an acute attack may present differently, render the diagnosis of AIP challenging. However, upon careful holistic assessment of patients’ presenting symptoms, in addition to ruling out other more common differential diagnoses, AIP should be considered. Thus, it is the overall clinical picture that must be considered in order to appreciate the systemic nature of AIP and therefore make its diagnosis.

In addition to recognizing classical features of an acute AIP attack, clinicians must also be aware of the varying phases of AIP to prevent an acute attack from progressing. In practice, however, this can be rather difficult: The initial prodromal phase of anxiety and restlessness describes nonspecific symptoms shared by multiple differential diagnoses, and patients may not necessarily present to the emergency department with these symptoms. Indeed, it is more likely that patients may present at the visceral symptom phase, which would delay their treatment. Nonetheless, regardless of the stage at which patients present, clinicians must be further educated on the varied symptoms and phases of an acute AIP attack so that they recognize the disease and intervene early.

Our review has also shown that the nonspecific presentation of AIP may lead to suboptimal treatment in the face of acute diseases. Patients’ symptoms may be interpreted as another disease, for which they may receive excessive treatment, while there may be negligence in falsely attributing new-onset symptoms as an acute AIP attack. Compared to other acute chronic diseases, such as a gout attack, which can be inferred clearly from a patient’s history, AIP poses a challenge in ruling in and ruling out other diseases during its acute presentation. In order to safely manage patients with AIP, hospitals must follow an evidence-based treatment algorithm that offers guidance in both symptomatology and management so that clinicians can order the appropriate investigations and initiate suitable treatment.

Management

Regarding the management and therapeutic approach of AIP, several clinical recommendations and practice guidelines do exist. In the following, we want to summarize the current state of evidence and review established clinical algorithms for the management of patients with AIP.

**Figure 3:**
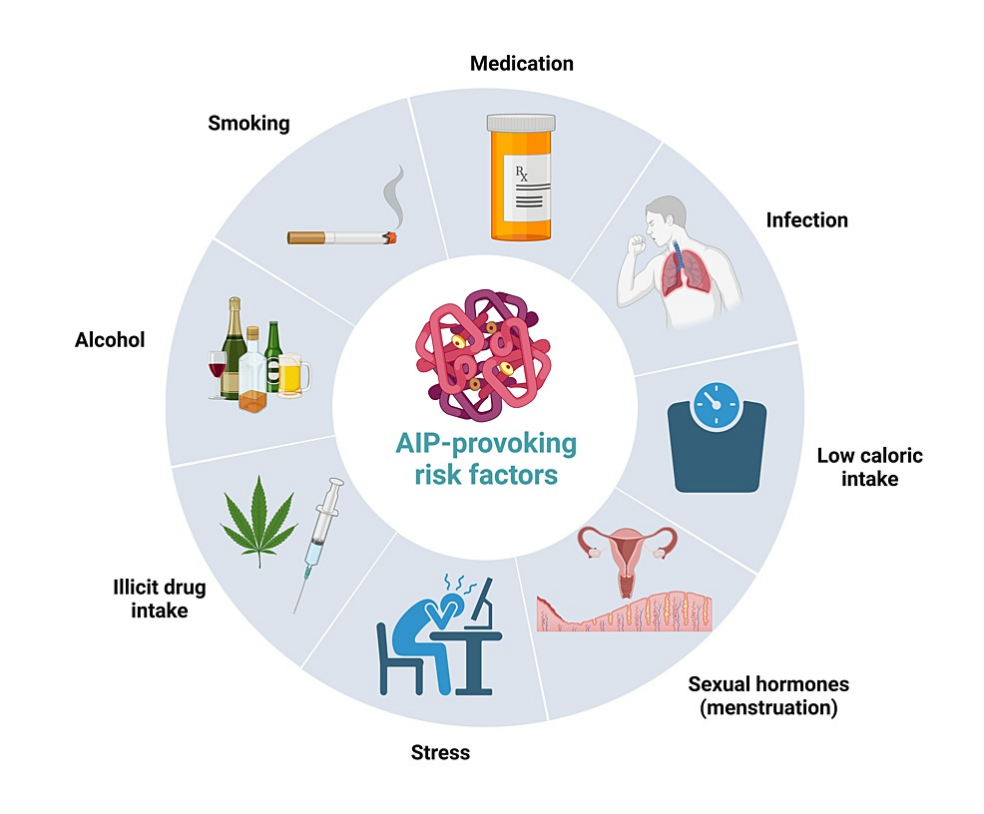
The most important precipitating factors for an acute intermittent porphyria attack. Adapted from “Risk Factors of Dementia,” created by Liv Nordestgaard using BioRender.com (2023). Retrieved from https://app.biorender.com/biorender-templates AIP: acute intermittent porphyria

In general, there are several factors to reconsider if suspecting an acute porphyria attack. The clinical presentation of the patient may give hints about the underlying condition. The most common clinical findings are abdominal pain, nausea, emesis, red urine, hypertension, tachycardia, proximal muscle weakness, and neuropsychiatric disorders. The exclusion of important differential diagnoses, such as appendicitis, cholecystolithiasis, or nephrolithiasis, strengthens the suspicion of AIP. Mostly, patients with a noticeable family health history presenting with similar symptoms or patients with former known and/or recurrent episodes of AIP are highly suspicious of developing recurrent episodes. Several risk factors may precipitate and actively provoke an acute porphyria attack, such as certain drugs (e.g., antibiotics, oral contraceptives, and anticonvulsants), sex hormones (especially with premenstrual symptoms), low calorie intake (fasting, dieting, or digestive disease), stress, infections, alcohol, smoking, or any other kinds of illicit drugs (Figure 3) [1,24,25]. Epidemiologically, AIP is more frequent in young female patients (66% have their first onset of AIP between 20 and 40 years of age) [7,26].

On principle, after AIP is suspected, urinary PBG analysis should be performed urgently. In general, quantitative measurements are preferred. The normal range for PBG in urine is around 0-4 mg/day. In AIP, urinary PBG can increase up to 20-200 mg/L [27].

Acute treatment should be started as fast as possible. Before starting treatment, urine, fecal, and blood samples for secondary testing should be derived, if this is the first porphyric attack of the patient and the diagnosis of AIP was not consolidated formerly. By determining erythrocyte porphobilinogen deaminase activity and plasma/urine/fecal porphyrin levels, a specific differential diagnosis of AIP, hereditary coproporphyria (HCP), and variegate porphyria (VP) can be performed later. Furthermore, subsequent gene sequencing and mutational analysis can be performed. But further differential diagnostic workup should not delay the acute management of the disease.

Regarding the concrete management, we divide it into acute management, treatment of concomitant symptoms, and long-term preventive treatment modalities.

Acute management

The treatment of an acute attack can be summarized into six main points (Table 1): hospitalization, discontinuing porphyrogenic agents, reducing ALA synthesis through hemin or glucose infusion, caloric support, correction and monitoring of electrolytes, and symptomatic treatment.

**Table 1:** Most important principles of acute AIP management. AIP, acute intermittent porphyria; IV, intravenous

	Acute Treatment of AIP
1.	Hospitalization
2.	Discontinuing porphyrogenic agents
3.	First line: hemin infusion (Panhematin 3-4 mg/kg IV for four days)/second line: glucose (3 L 10% glucose/24 hours)
4.	Adequate caloric support
5.	Correction and monitoring of electrolytes
6.	Treatment of symptoms

​​​​​​In all patients with mild or severe AIP, it is recommended to use heme or heme arginate derivates as first-line therapeutic approach. Heme administration suppresses the production of porphyrins and their precursors [24,25]. Application should take place urgently.

In the United States, only the FDA-approved Panhematin is available. In Europe, mostly heme arginate derivates are available.

For practitioners in the United States, it is recommended, in concomitance with the guidelines of the American Porphyria Association, to apply Panhematin 3-4 mg/kg running over one hour intravenously [27]. Panhematin is a lyophilized drug, so it is necessary to solve the freeze-dried powder first. Therefore, it is suggested to solve Panhematin with albumin 20% instead of sterile water. With a concentration of 20%, it is possible to construct a nearly 1:1 dilution of albumin and hematin. With that, a proper binding of hematin onto albumin is more probable to prevent common adverse effects, such as phlebitis [24].

Because there is a risk of developing phlebitis with possible loss of superficial veins, it is recommended to only apply Panhematin into a large vein or through a central venous catheter. After Panhematin has run through, rinsing with 250 mL 0.9% NaCl solution should be performed, to clean out remnants of hematin.

For practitioners based in Europe or Africa, it is approved to use heme arginate (Normosang®) 3 mg/kg, maximal dosage 250 mg once daily for four days, in concordance with the practice guidelines of the National Health Service’s (NHS) Specialist Pharmacy Service [28].

As second-line treatment option, 3 L of glucose 10%/24 hours may be applied. This treatment should only be reserved for mild cases (mild pain, which is manageable with non-opioid analgetic, no hyponatremia, and no neurovisceral symptoms). Glucose treatment has been observed to be less effective than hematin in several studies [29]. Furthermore, there is a risk of inducing or worsening hyponatremia due to dilution [1]. Carbohydrate loading was the mainline of treatment before the availability of hemin and is still used, when hemin is not readily available [25].

Most patients react promptly to treatment, so clinical remission is common in 24-48 hours. Of course, any precipitating factors (mentioned above) should be avoided. There is a multitude of drugs that can provoke AIP. For more detailed information, we recommend the use of the drug database of the American Porphyria Association [30] and consulting an experienced physician of the field.

Treatment of symptoms

AIP presents with a multitude of different symptoms. In order to ensure the best standard of quality, a symptomatic treatment approach should be included into management.

Gastrointestinal Symptoms

Over 80% of patients present with acute abdominal pain,the most common symptom of AIP [1,29,31,32]. Analgetic management should follow the WHO guidelines. In rarer cases of mild pain, nonsteroidal anti-inflammatory drugs (NSAIDs) or other non-opioid analgesics may be sufficient [25]. Most of the patients present with intense pain (visual rating scale {VRS} > 7) [8,9], so the management with opioids should be preferred. Morphine and buprenorphine are the first-line opioid agents for the management of pain [33]. Some analgesics (fentanyl, tramadol, nalbuphine, oxycodone, and hydrocodone) have shown in some studies to elevate porphyrin levels and should be avoided [25,34]. Gabapentin and opiate patches are helpful in the treatment of neuropathic pain [33,35]. Nausea and vomitingmay* *be treated with chlorpromazine, droperidol, prochlorperazine, or ondansetron [33]. For ileus,* *neostigmine may be beneficial [11,33]. Dysphagia can sometimes occur, which can be treated with nasogastric (NG) tube for nutrition [33].

Neurological Symptoms

For the treatment of seizures, it is recommended to avoid carbamazepine, phenytoin, and valproic acid as these are mainly metabolized by the liver [36]. The recommendation is to use anti-epileptics, which are not metabolized by the liver, especially levetiracetam, gabapentin, topiramate, and lacosamide [25]. Drugs such as benzodiazepines are safe [25].

The management of SIADH should follow regional guidelines. In milder cases with euvolemic hyponatremia, solely fluid restriction to 1 L/day may be enough. In moderate to severe cases of hyponatremia (Na+ < 125 mmol/L), intravenous (IV) 3% saline solution bolus of 100-150 mL should be applied over 10-20 minutes until Na+ has increased over 5 mEq/L [9]. Electrolyte levels should be regularly controlled.

Psychiatric Symptoms

Psychiatric symptoms, such as anxiety, depression, schizophrenia, and hallucinations, can be managed with short-acting benzodiazepines and chlorpromazine. Fluoxetine can be used for depressive symptoms [25]. Fluoxetine can be applied against depression and is the only selective serotonin reuptake inhibitor (SSRI) that is not provoking porphyric attacks [25]. 

Others

ECG should be performed, if arrythmia occurs. Serum electrolytes should be controlled in regular intervals. Tachycardia and hypertension can be treated with beta-blockers, calcium antagonists, or angiotensin-converting enzyme (ACE) inhibitors [11,25,33]. If progressive paralysis with impeding respiratory distress occurs, admittance to the ICU, tight monitoring, and mechanical ventilation may be necessary [3]. Early physical therapy and occupational therapy are recommended for the management of muscle weakness [11,33]. Patients can sometimes have bladder paresis for which Foley’s placement is required [33].

Long-term/preventive management

A small minority of patients can have recurrent attacks with AIP [24]. For long-term preventive management, the most important approach is to keep abstinence of provoking factors.

Sexual hormones are known for precipitating API attacks. Approximately up to 1/3 of patients present with symptoms during premenstrual stage [25,37]. Progesterone is suspected to play a role as an inducer of ALAS1, which leads to the increased accumulation of toxic metabolites [25]. If recurrent premenstrual porphyric attacks are observed in a female, gonadotropin-releasing hormone (GnRH) analogs can be used, such as buserelin or leuprorelin. The therapy should be started 1-3 days after the beginning of menstrual cycle so that an acute AIP crisis can be avoided, based on partial agonist activity of GnRH analogs with probable hormone increase [24,25]. If using medication over six months is planned, accurate prevention and monitoring for osteoporosis and endometric dysplasia should occur. Other signs of estrogen deficiency, such as flushes, may be possible.

In some cases, long-term heme arginate therapies could be beneficial too [24,25,33]. Mostly, if the patient is having multiple and not regular cyclic attacks over the year, which do not respond to medication, long-term therapy may be performed. Once a week hematin of 3-4 mg/kg may be administered. In long term, hematin may cause iron overload. Blood tests should be performed regularly; if necessary, phlebotomy should be done.

Next to that, new and promising therapeutical approaches have been developed for the treatment of AIP over the last few years.

**Figure 4:**
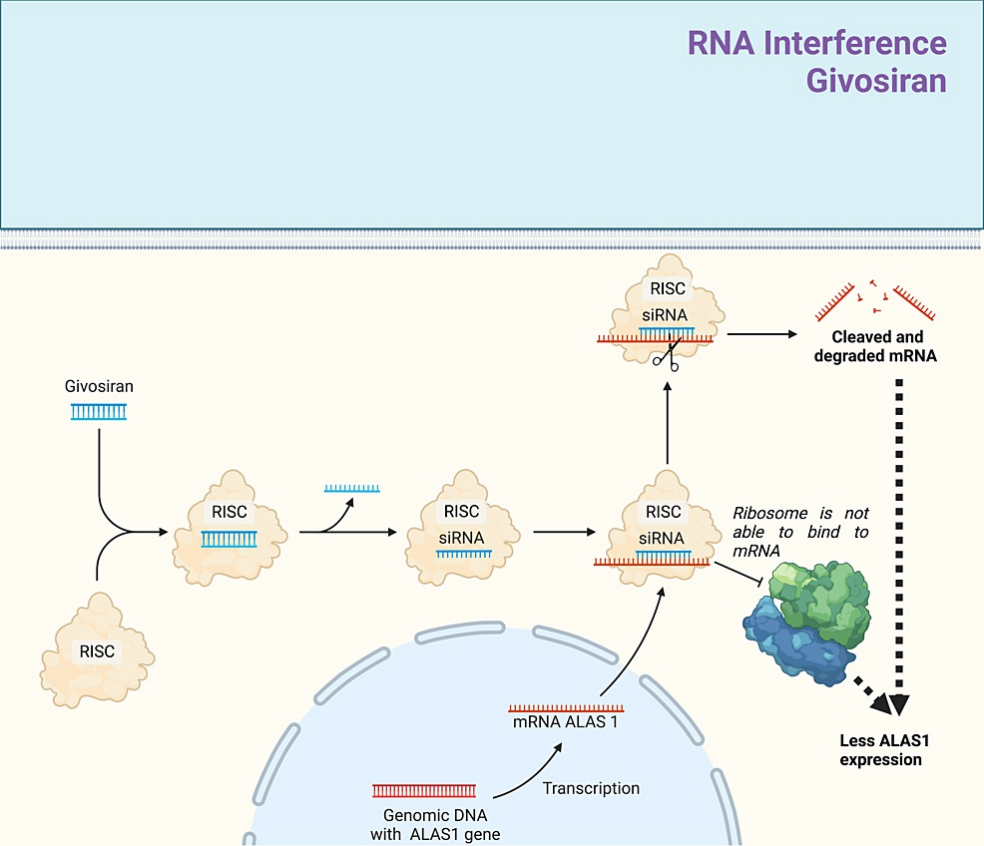
Overview of the mode of action of givosiran in the long-term treatment of AIP. Givosiran is FDA-approved since 2019 for the long-term treatment and prevention of recurrent AIP attacks. Givosiran is a small interfering (si) RNA molecule. After transportation into the intracellular compartment, it first gets hydrolyzed into a single-strand molecule by reacting with the protein complex RNA-induced silencing complex (RISC). Bound onto the RISC, the single-stranded siRNA can bind to messenger (m) RNAs with a similar complementary base pair (bp) sequence. Givosiran has a high affinity for binding onto the messenger (m) RNAs derived of *ALAS1* gene. By binding onto ALAS1 mRNA, the ribosomes cannot interact sterically with the mRNA anymore, diminishing the gene expression of ALAS1 enzyme. If there are many differences between the base pair sequence of siRNA and mRNA (ALAS1), givosiran may induce mRNA degradation through RNA interference, leading to a breakdown of mRNA. By reducing the expression levels of ALA1 enzyme, the buildup of aminolevulinic acid during heme synthesis will be reduced too. Consequently, the amount of toxic metabolites, such as ALA and PBG, will be reduced, and with that, clinical management may be possible. Adapted from “siRNA Nanoparticle Delivery System,” using BioRender.com (2023). Retrieved from https://app.biorender.com/biorender-templates AIP, acute intermittent porphyria; ALA, aminolevulinic acid; ALAS1, aminolevulinic acid synthase-1; PBG, porphobilinogen

Givosiran is a drug utilizing the effects of RNA interference as small interfering (si) RNA (Figure 4). It is FDA-approved since 2019 and since 2020 available in Europe. It is indicated for patients with AIP to prevent further attacks [31]. Through RNA interference, givosiran degrades and prevents further translation of the mRNA of ALAS1. ALAS1 is an enzyme that produces ALA and enables PBG production, the toxic products that are responsible for AIP manifestation. By reducing the amount of enzyme, the amount of toxic metabolites is consequently reduced too. Givosiran is not a part of the American guidelines yet [27]. However, the use of givosiran is recommend in the recent BMJ best practice guidelines [38]. We recommend applying the standard dosage of 2.5 mg/kg adjusted to bodyweight per month, as recommend by the manufacturer [38,39].

As a final possibility, especially in patients with recurrent attacks, who do not respond to medication, orthotopic liver transplantation (OLT) may be recommended [40,41].

Emerging therapeutic innovations

Besides the aforementioned established treatment options, there are several newly emerging innovative treatment modalities, which are part of experimental studies. In this review, we will mainly focus on recombinant enzyme replacement and adeno-associated virus (AAV)-mediated gene therapy (GT) and explain the role of chaperones and (PBGD) mRNA [42].

One very promising and frequently discussed idea is the application of PBGD messenger (m) RNA [42]. mRNAs are small single-stranded molecules of RNA, which are used by ribosomes as templates for protein synthesis. In AIP, different mutations lead to a decreased production and function of endogenous PBGD (also called as HMBS), which cause an accumulation of ALA and PBG and with that clinical manifestations. To increase the function and availability of PBGD, it may be possible to “deliver” the cell with physiologic PBGD mRNA externally. The cells are able to internalize PBGD mRNA, if it is carried in the form of nanoliposomes [42]. This idea was tested out by a study group on mice and nonhuman primate collectives with very promising results [42,43].

Another field of current research is adeno-associated virus (AAV)-mediated gene therapy (GT) [42]. The goal of gene therapy is to create a long-term solution for the disease by directly focusing on where the pathology of AIP arises. AIP is caused by the inheritance of mutations in the genes coding for PBGD/HMBS. The concept of gene therapy is to recreate the physiologic gene sequence by inserting the gene through vectors with the help of a liver-specific promoter in the genome of the patient. In one mice trial, vector rAAV2/5 encoding human PBGD cDNA could be inserted by the help of promoter (rAAV2/5-PBGD) [42,44]. It was observed that the PBGD enzyme function was reestablished and porphyrin precursor buildup was decreased [44]. Because gene therapy is a long-term intervention, it could be especially beneficial for patients who are suffering under this condition frequently through severe attacks. Nevertheless, clinical trials on humans are necessary in order to check safety, side effects, drug dosage interactions, and clinical efficacy. At the moment, phase I clinical trials on humans are conducted. One of the studies revealed drug safety with only minor side effects [42,45]. A decrease in hospitalization rates and the necessity of heme treatment was observed too [42,45]. Nevertheless, there was no significant reduction of ALA and PBG levels, so a higher dose application of the drug may be necessary [42,45].

Recombinant enzyme replacement therapy is another important concept. Here, the idea is to apply a physiologic working enzyme replacement externally, e.g., through subcutaneous injection. The efficacy of this method needs to be further clarified. Some studies on human collective have shown no promising effect in acute attacks, and the enzymes got quickly deactivated in the peripheral circulation [42,46]. Currently, there are trials to adapt the recombinant enzyme, in order to increase its stability and efficacy. One study tried out a linkage of the enzyme with apolipoprotein AI (rApoAI-PBGD) in a mouse model and has shown decreased acute attacks [42,47]. Furthermore, the half-life could be increased from 45 minutes to approximately 10 hours with persisting serum levels of up to six days [42,47]. There is still a need to find further methods to increase the stability and activity of recombinant enzymes. Also, these adapted versions of recombinant enzymes still do need verification in human studies.

Finally, there is the idea of using chaperones [42]. Chaperones are small hexagonal molecules that are used in order to stabilize and increase the half-life of otherwise unstable, easily degradable (mutated) enzymes. By using a chaperone, which is specifically designed to bind and stabilize PBGD, the half-life of PBGD can be increased, which may lead to an increased duration of enzyme activity. Animal studies have already shown benefits, such as increased PBGD activity and decreased amounts of porphyric precursor accumulation [42,48]. The limitations of this concept are that probably, a partial residual activity of the PBGD enzyme is necessary. If a PBGD mutation is causing a complete loss of function, stabilizing the enzyme through chaperones would not be very beneficial. Furthermore, these advances need further verification through experimental studies on human cohorts.

In general, several new therapeutical approaches, such as gene therapy, enzyme replacement therapy, PBGD mRNA, chaperones, and *ALAS1* gene inhibition, are currently the focus of research and may be possible treatment options in the future [18,25].

## Conclusions

AIP has a complex and nonspecific presentation with abdominal pain, nausea, and vomiting being the most common presenting symptoms. It can involve the cardiovascular, renal, endocrine, and nervous systems with psychiatric manifestations, among others. The acute management of AIP is heme/glucose administration and supportive treatment of symptoms. Preventive treatment is the mainstay of AIP management, and patients should be counseled about how certain risk factors can trigger their disease symptoms. Besides a few new treatment modalities that target at a cellular level to control the enzyme ALAS1’s activity, there is promising research ongoing that has the potential to revolutionize the treatment and long-term management of AIP.
